# The European Health Data Space in communicable diseases surveillance and monitoring of medicines and vaccines: achievements of a pilot project examining the user journey

**DOI:** 10.1093/eurpub/ckaf115

**Published:** 2025-09-10

**Authors:** Katharina L Schneider, Luís Alves de Sousa, Ionut Sava, Steffen Heß, Denise Umuhire, Daniel R Morales

**Affiliations:** Data Access and Coordination Office, Federal Institute for Drugs and Medical Devices, Bonn, Germany; European Centre for Disease Prevention and Control, Stockholm, Sweden; European Centre for Disease Prevention and Control, Stockholm, Sweden; Health Data Lab, Federal Institute for Drugs and Medical Devices, Bonn, Germany; Real World Evidence, The Data Analytics Taskforce, European Medicines Agency, Amsterdam, The Netherlands; Real World Evidence, The Data Analytics Taskforce, European Medicines Agency, Amsterdam, The Netherlands

## Abstract

The future European Health Data Space (EHDS), a network for secure cross-border data use, could be beneficial for public health initiatives. The HealthData@EU pilot project evaluated possibilities of secondary data use based on five use cases and established a pilot IT infrastructure. This article reports overarching experiences from two public health use cases and the IT development. Experiences were reported by the Users’ Journey steps (data discovery, application, use, and finalization) elaborated in the context of the European Health Data Space (TEHDAS), the first conceptual EHDS project. The European Medicines Agency’s use case analysed coagulopathy-related events in COVID-19 patients, the one led by the European Centre for Disease Prevention and Control assessed the feasibility of HealthData@EU to support antimicrobial resistance surveillance. The IT work package developed the infrastructure for information exchange. The use cases indicated that standardized procedures, wherever available, were considered highly beneficial. In the application phase, node-specific requirements slowed down the progress. A distributed approach in combination with the use of Secure Processing Environments (SPE) was successfully conducted. The IT infrastructure was piloted, tested, and published as open source. It supports data discovery and applications in the future. The HealthData@EU pilot project has achieved major steps regarding data discoverability and accessibility, where a need for more standardized procedures was detected in the use cases. Distributed analyses in combination with SPE use may be possible approaches for the data use phase, which require further investigation in TEHDAS2 and other initiatives.

## Introduction

Real-world data are increasingly important in complementing data from randomized controlled trials. Large datasets representative of the general population can answer a variety of research questions ranging from natural disease epidemiology, health care utilization, and aetiological comparative cohort studies, for example. Over recent years, several initiatives have been developed in Europe to further investigate the use and value of real-world data to support healthcare and policy decision making. The European Regulatory Network has published an operational, technical, and methodological framework for the regulatory use of real-world evidence consisting of 11 workstreams including the establishment of the Heads of Medicines Agencies (HMA)/European Medicines Agency (EMA) Data Analysis and Real World Interrogation Network (DARWIN EU) [1–3]. In short, DARWIN EU is the EMA’s distributed network of data, expertise and services, orchestrated by a coordination centre working with data partners that aims to support regulatory decision making with real-world-evidence.

The European Centre for Disease Prevention and Control (ECDC) collects, validates, analyses, and disseminates routine surveillance data on notifiable communicable diseases and related special health issues from 30 European Union (EU)/European Economic Area (EEA) countries. ECDC is mandated to strengthen digitalization and integration of surveillance systems to most timely and effectively address cross-border threats to public health [4–6]. Antimicrobial resistance (AMR) is one of the most pressing public health challenges requiring efficient surveillance processes and consistent and timely data for evidence-based interventions [7].

Both the EMA and ECDC are facing urgent need for high quality real-world data and aim to address current gaps in their availability across a scattered data landscape. The European Health Data Space (EHDS) sets out a common EU framework that allows for use of health data for research, innovation, public health, policy-making, regulatory activities and personalized medicine by creating a unified health data infrastructure [8]. The Joint Action Towards the European Health Data Space (TEHDAS) was the project laying the concept groundwork for secondary use of health data under the EHDS and included a description of the TEHDAS users’ journey: data discovery, data permit application, data use, and project finalization phase [9, 10]. This concept has been a basis for the HealthData@EU pilot project. The scope of the pilot project was to build a first version of the future EHDS infrastructure [11]. It consisted of four horizontal work packages, five technical work packages as well as five research use cases. EMA and ECDC were actively involved and provided two of these use cases which represented good examples for future benefits of the EHDS for public health (‘Assess the feasibility of the EHDS framework for secondary use to support antimicrobial resistance surveillance’ led by ECDC, and ‘Natural history of coagulopathy (blood clotting)-related events in COVID-19 patients and risk factors’ led by EMA). Methods and results of their quantitative analyses were not within the scope of this article and the work of other use cases and work packages is published elsewhere. This article aims to describe their experiences with a specific perspective on health monitoring in Europe and the achievements of work package (WP) 5 ‘IT infrastructure’ that may in the future turn the learnings into reality.

## Methods

### Experiences along the user’s journey

Experiences and outcomes from HealthData@EU WP5 and the two use cases were collected from leads and data partners, and structured based on the TEHDAS users’ journey published in deliverable 7.2 on services and infrastructure for secondary data use in the EHDS [10]: data discovery, permit application, data use and finalization phase. Each learning or outcome was allocated to one of these steps. The objective was to directly match detected gaps with the opportunities of the future infrastructure. Definitions of relevant terms are available in Supplementary Material S1.

### HealthData@EU work package 5

Work package 5 ‘IT infrastructure’ was co-led by the French Health Data Hub and German Health Data Lab [12, 13]. The timeline consisted of four phases: framing, proof of concept (POC), minimum viable product (MVP) and deployment. WP5 was divided into five workstreams. In the governance workstream, a governance model was established, work packages planned and POC nodes were identified. Also, a framework for development and deployment was formulated. The final documents for the target operating model and the architecture definition are being prepared at the time of this writing. In the functional workstream and the technical workstream, the respective functional and technical requirements for the infrastructure were compiled and a framing note was written before beginning the actual development which was finalized in June 2024. The security workstream entailed an initial collection of the security requirements and a security risk analysis. The change management workstream addressed the communication of the proof of concept and the deployment of the POC nodes that tested the infrastructure [14].

### Summary of the EMA use case

The EMA use case examined coagulopathy related events in COVID-19 patients and risk factors. The EMA led the design and coordination of the use case and contributed data partners through the EMA DARWIN EU network, namely CPRD (UK), Estonia Biobank (Estonia), IPCI (Netherlands), SIDIAP (Spain), and IQVIA Germany (Germany). Outside DARWIN EU, additional data users and holders included: The Finnish Institute for Health and Welfare (THL) using data from the national Care Register for Health Care (Hilmo); the Croatian Institute of Public Health using data from the National Public Health Information System (NAJS), the French Health Data Hub accessing data from the Système National des Données de Santé (SNDS); and the Danish Medicines Agency’s Data analytics group using Danish health data registers.

The EMA use case had five objectives of growing complexity focused on the incidence of venous and arterial thromboembolic events in relation to COVID-19 during the Omicron SARs-CoV-2 period. A distributed analysis study approach was undertaken using a common protocol developed by the consortium (EUPAS107315) [15]. Except for DARWIN EU, which used the Observational Medical Outcomes Partnership common data model (CDM), each node could choose to perform analysis in their native source data or convert their data to OMOP CDM format [16].

The EMA use case tested each nodes’ ability and approaches to identify, access and link complex health data from different registers (e.g. SARs-CoV-2 vaccinations); the infrastructure for processing and analysing large cohorts; and their capacity to analyse native data or map it to OMOP CDM. It also compared DARWIN EU to other nodes’ processes and the interoperability of study packages to data nodes outside of the network.

### Summary of the ECDC use case

The ECDC use case compared carrying out AMR surveillance under a test distributed framework (individual analyses for each data source and final pooling of the results) with the historical reference of centralized AMR surveillance performed by ECDC. Objectives were: (i) to test distributed technical solutions for AMR surveillance data sharing between national nodes and ECDC; (ii) to identify legal challenges of data use under the pilot; (iii) to assess concordance between AMR surveillance data shared through the use case and data previously submitted to ECDC through the European Surveillance System (TESSy), the epidemiological surveillance platform operated by ECDC [17], for collecting communicable diseases surveillance data from EU/EEA countries.

Participating institutions included: The Belgium Institute for Health (Sciensano) as data holder and user; the University Hospital for Infectious Diseases Dr Fran Mihaljevic (BFM) as data holder and the Croatian Institute of Public Health (HZJZ) as user; the Finnish Institute for Health and Welfare (THL) as data holder and user; and the Norwegian Institute of Public Health (FHI) as data holder and the Norwegian Directorate of eHealth (ehelse) as user.

The AMR surveillance data for 2020, according to the European Antimicrobial Resistance Surveillance Network (EARS-Net) metadata, were used as the reference dataset. The participating nodes were required to retrieve this dataset according to the specific EARS-Net reporting protocol [18]. The analysis followed the EARS-Net common analysis plan [19]. The EARS-Net AMR surveillance report for the yearly surveillance period of 2020, including AMR case counts and specific indicators of AMR, was used as the historical reference [20].

## Results

### Data discovery phase

#### EMA use case

**Figure 1.**
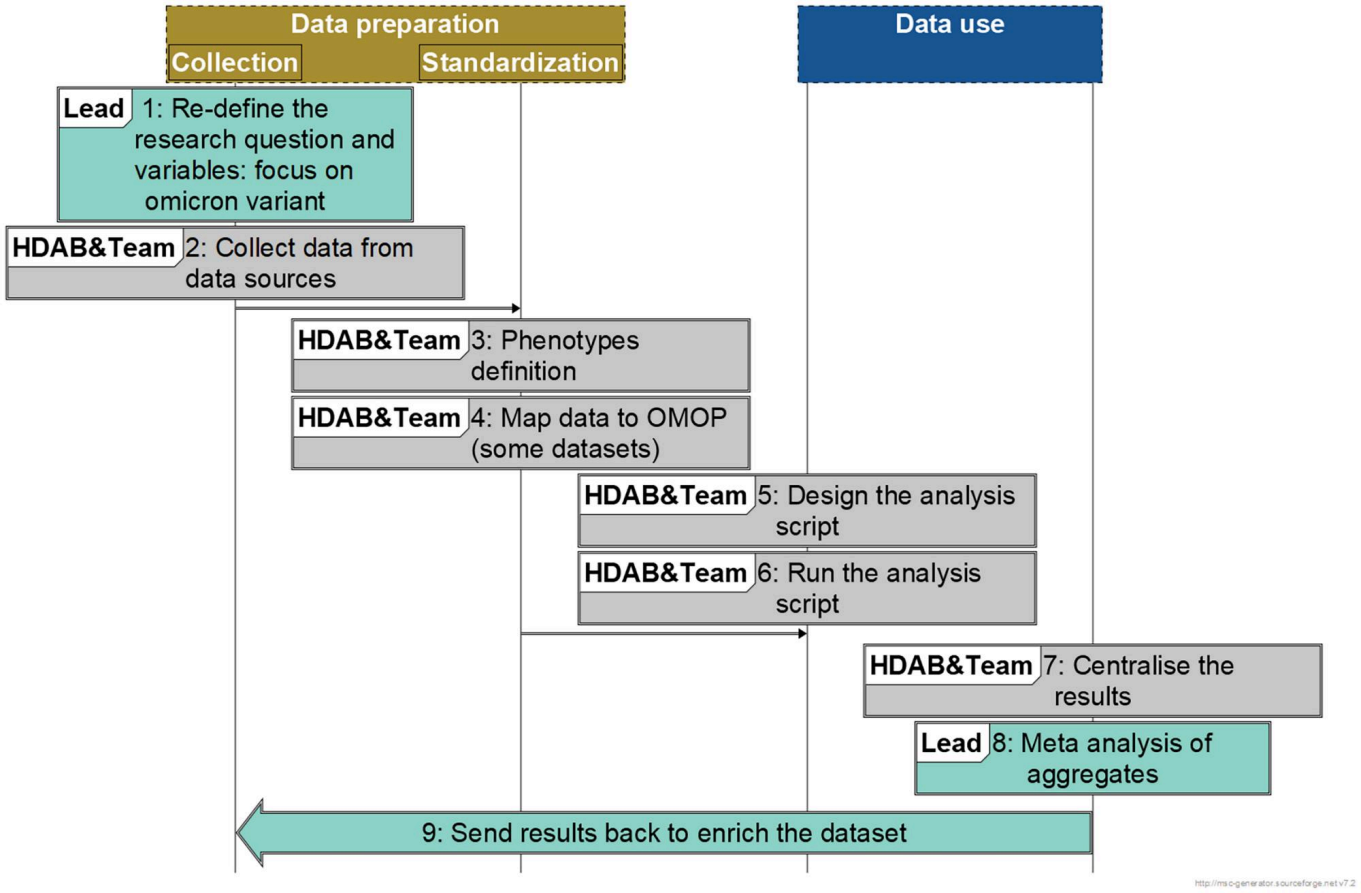
Diagram showing the steps of the EMA use case (UC) journey.

The EMA user journey is summarized in Fig. 1. DARWIN EU used a structured feasibility assessment to identify relevant data sources. It used metadata from HMA-EMA catalogues. For other data nodes, data discovery relied upon existing local expert knowledge in an unstructured manner and no metadata catalogue was used. The DARWIN EU process provided clarity before the start of the study on the limitations of each database for answering the objectives. For example, of the five selected DARWIN EU databases, only one contained data to address all five study objectives, whilst three could address four objectives, and one was used to address one objective.

#### ECDC use case

**Figure 2.**
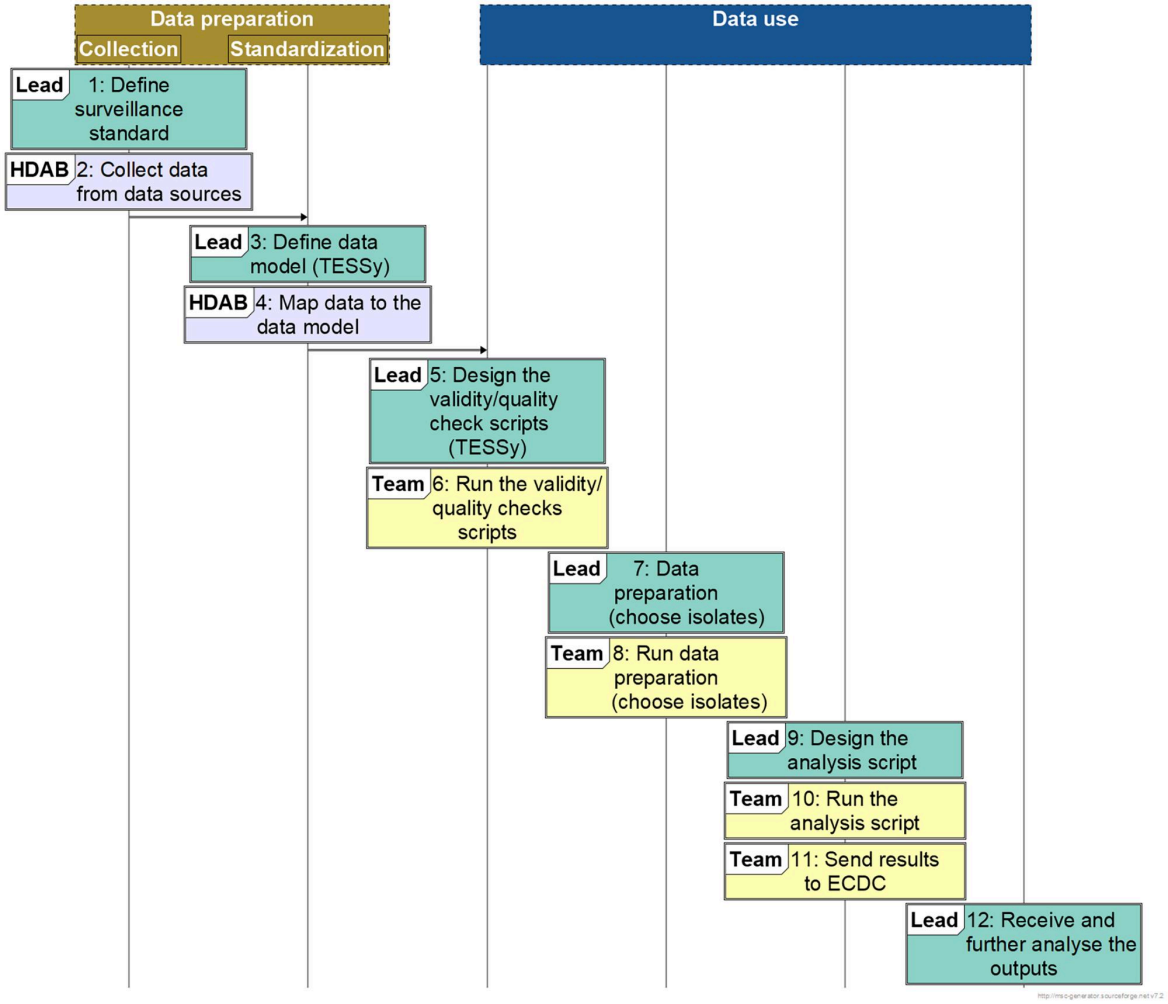
Diagram showing the steps of the ECDC use case (UC).

The ECDC user journey is shown in Fig. 2. Data discovery relied on existing practices for AMR surveillance at national level alongside the coordination and support provided by the EARS-Net network, as well as the common EARS-Net reporting protocol. This protocol contains a metadata description, which has been used for data submission to TESSy for several years. Data discovery also relied on existing knowledge of national data sources.

The Norwegian node withdrew from the use case, as there were national-level legal constraints to the data retrieval, impeding the transfer from the data holder to the node.

#### Work package 5

The objective was to build a secure technical infrastructure for data exchange between national nodes and the European Commission via eDelivery. It was designed agnostically to support diverse EHDS use cases. The architecture consists of Cross-Border Gateways for the eDelivery communication, National Connectors that provide the features for the user journey, and Cross-Border Engines that help the national nodes extend their current functionalities for EHDS tasks.

The EHDS regulation foresees that Health Data Access Bodies publish a national metadata catalogue connected to a central EU metadata catalogue. This connection can be established via the Cross-Border Gateway. In concrete terms, the Cross-Border Engine can identify updates within the national metadata catalogue and this information reaches the EU metadata catalogue via the National Connector, Cross-Border Gateway, and EU Connector. This infrastructure also entails feedback to the national Cross-Border Engine containing the status of the operation. Services, therefore, enable creation, update and deletion of datasets in the EU Dataset Catalogue and restoration of a catalogue. The code is open source [21]. Deployment and testing of POC nodes infrastructure was successful.

### Data permit application phase

#### EMA use case

Approval and data access time ranged from 1 to 12 months for nodes and 1–4 months for DARWIN EU. Except for one, databases required Internal Review Board or scientific committee approval using new protocols to conduct the study.

Data nodes needed to submit a permit application in the native language, with varying requirements and timelines. In Finland, while the research team had access to the required datasets from a previous COVID-19 mandate, a new authorization for reuse was required. In France, a detailed research application process was required, leading to additional specifications in the agreed protocol and access requests assessed by two different committees sequentially. This significantly delayed approval and required weeks of work from local teams. In contrast, Croatia used an existing template reviewed by one board, with approval in under 6 months.

Some databases did not require data minimization, others required data minimization related to the range of variables, and some required both a reduction in the range of variables, datasets, and approved only the use of a sample of the available pseudonymized population.

#### ECDC use case

The data application process primarily depended on country-specific legal and organizational factors. A data processing and transfer agreement had to be concluded between ECDC and each node. This was a time-consuming step as provisions had to be tailored to the context of the use case, national legal requirements, as well as reflecting the different types of parties involved. There was no existing framework for cooperation in decision making, which slowed down the progress. Overall, ∼1 year was required to complete all legal proceedings for all nodes.

Data protection requirements also varied. For example, the definitions of data controller and processor were debated with different nodes taking different approaches and required the consultation of data protection authorities by both parties to ensure compliance.

#### Work package 5

The data permit application process was the second use case for the new IT infrastructure. The HealthData@EU infrastructure foresees the possibility for a user to apply for the data by filling out a central EU application form provided by the central services. This application is sent via the EU connector and the Cross-Border Gateways to the National Connector that stores the application in a database. Afterwards, it can be imported into the National Data Permit Management System by the Cross-Border Engine. The system also supports application status updates. The code is open source [21]. The deployment and the testing of the POC nodes infrastructure was successful.

### Data use phase

#### EMA use case

Data were processed locally by each data node using their own Secure Processing Environment (SPE). Databases within DARWIN EU contained OMOP CDM-mapped data. Other nodes performed OMOP CDM mapping voluntarily. Reasons for mapping were related to increasing interoperability with harmonized variables and preferring to execute the pre-prepared DARWIN-EU analytical script.

The use case involved large, vaccinated cohorts covering most of the database population. This created challenges in OMOP data analysis due to infrastructure constraints. This was resolved by improving computer hardware systems to increase memory, amending the analytical R package to process and save results of study objectives in a stepwise manner, and using random samples of the population. Other challenges included limited experience of data nodes in executing standardized analytics using the OMOP CDM in their SPE, which had different specifications requiring additional support.

#### ECDC use case

Each node created or acquired SPE services specifically for this use case. For the nodes in Belgium and Croatia, a dedicated SPE was prepared on each institutional premise. For the node in Finland, an SPE service was provided, according to dedicated national legislation [REF[LA1]].

The analysis package, including the programming code for data validation, cleaning, and epidemiological indicator computation, was delivered beforehand with each participating node for forensic and technical review. This package was used both by node and ECDC staff to carry out the analysis, which was done in a distributed approach in two stages. Initially, a node-specific SPE was used to hold the required country-level AMR surveillance dataset of 2020 as per the EARS-Net protocol [18]. A national-level analysis was performed by node staff (i.e. only the analysis code visited the data in the SPE) and by ECDC staff (i.e. data user and analysis entered the SPE), respectively. Subsequently, an EU/EEA-level pooled analysis was carried out by ECDC staff at a test central SPE, held by ECDC, to which the three datasets had been transferred. This was important to assess a pooling SPE for scenarios where distributed approaches are not feasible [18, 22].

#### Work package 5

The data use phase was outside the scope of work package 5. The HealthData@EU infrastructure foresees that data will be provided and processed in Secure Processing Environments (as described in the use cases) that increase data protection as they do not allow any copying or downloading by the user. SPEs are a key focus of TEHDAS2, the TEHDAS follow-up project launched in 2024. Work package 7, co-led by the same team as WP5 of the pilot project, will develop SPE technical specifications and user guidelines. The aim is to support future implementing acts for the EHDS.

### Finalization phase

#### EMA use case

Anonymized aggregated results were generated as counts that could be shared between research teams, but no further processing was conducted. Within DARWIN EU, aggregated results from all 5 databases were shared with the DARWIN EU coordination centre that prepared a study report that was published in the HMA-EMA Catalogue of real-world data sources and studies [15]. Other nodes generated, reviewed, and interpreted results both locally and centrally. To date, three data nodes are still executing the analytical package, and one data node is still performing OMOP CMD mapping after approximately 2 years. In contrast, the DARWIN EU study and native data analysis in Croatia have already been completed. No meta-analysis was performed due to result heterogeneity and governance rules requiring obfuscation of small cell counts. All nodes still store data locally in their SPEs while executing the analysis.

#### ECDC use case

The analysis package defined standard tabular outputs. These tables contained different epidemiological indicators, in accordance with the established yearly analysis of AMR surveillance data for EARS-Net. All nodes exported output files within 2 weeks. Aggregate-level indicator results for AMR were used to assess the concordance of the use case analysis, including the distributed analysis and pooling, by comparison with historical values, published in the AMR EARS-Net Report for 2020 [18]. Overall, concordance for AMR case counts was 100% (Belgium, *n* = 4320), 99% (Croatia, 2212/2017), and 80% (Finland, 8029/10052). Conditional on the publication of the HealthData@EU pilot and ECDC’ use case report, the AMR surveillance data held in all SPEs was deleted by the end of the pilot project.

#### Work package 5

The data finalization phase was outside the scope of work package 5. HealthData@EU foresees SPE analysis results being exportable in statistical format, as described in the use cases. Export and archiving questions will be addressed in TEHDAS2 work package 7. The topic of publication of the results will be discussed in work package 8 ‘Serving citizens’ where guidelines for users on handling research outcomes will be elaborated.

## Discussion

The EMA and ECDC use cases highlighted key challenges in the secondary use of health data. They also demonstrated the potential of distributed analyses while IT infrastructure was developed to address challenges in data findability and application. This article presented the experiences and achievements of the use cases and IT work package along the TEHDAS user journey. The data discovery phase is the crucial first step of the process and is also included in the widely accepted FAIR principles [23]. Both use cases highlighted the advantages of established infrastructures complemented with local expertise (e.g. specific metadata located at the data holder). The EHDS aims to improve data findability through standardized metadata catalogues at national and EU level. For this purpose, the Health DCAT-AP extension has been developed in the pilot project [24] which will be used to create an EU catalogue interoperable with the EMA metadata catalogue, for example. The IT infrastructure work package provided a technical solution to exchange these metadata between the national nodes and the EU central services. This could significantly improve the visibility of data sources for ECDC, EMA, and other stakeholders.

In the application phase, both use cases faced varying national requirements and timelines, with some processes exceeding 1 year. Such delays hinder timely responses to public health questions. Unpredictable timelines also complicate project planning and execution. In this regard, existing application templates proved beneficial. One main EHDS goal is to harmonize these processes. A common data application form was elaborated in the pilot project [25]. The network infrastructure for application exchange was developed by work package 5, aiming to facilitate a one-stop application process that enables EU central services to efficiently forward applications to national Health Data Access Bodies. Once operational, this will improve data accessibility and advance adherence to the FAIR principles [23].

A distributed approach was used for the analyses in both use cases. Whilst this approach has advantages in terms of data privacy, as data remain in the local SPE, it did not accelerate approval timelines. In this context, the EMA use case also included the approach using the OMOP CDM which may facilitate distributed analyses. The ECDC use case tested pooling analysis with a central SPE, additionally to local SPE use. Just as in the use cases, the EHDS regulation foresees the use of SPEs and the Health Data Access Bodies will play a crucial role in coordinating projects that require options for distributed analyses by supporting the communication between all actors involved. Technical solutions for SPEs exist in several Member States. However, this topic was beyond the scope of the pilot project and will be addressed in TEHDAS2.

The finalization phase has not been completed for the EMA use case (only for DARWIN EU), whilst the ECDC use case has been completed. Apart from publications in scientific journals, some data nodes have existing solutions in place where the research results are publicly displayed. This is also foreseen in the EHDS regulation and addressed in TEHDAS2 to ensure transparency, which can have an important impact on public trust in the EHDS.

In summary, the EMA and ECDC use cases highlighted the benefits of leveraging existing infrastructures, wherever possible. However, in many cases there were node or country-specific requirements that slowed down the progress, in particular in the application phase. The future HealthData@EU infrastructure aims to overcome these challenges, and the pilot project was a major step in this direction. The outcomes of this project will be leveraged in current and future initiatives such as the direct grant projects for HDAB establishment and TEHDAS2.

## Supplementary Material

ckaf115_Supplementary_Data

## Data Availability

This article focuses on the evaluation of processes and does not report results of statistical data analyses. No new data were generated or analysed in support of this research. Key points The use cases of the HealthData@EU pilot project led by EMA and ECDC indicated that node or country-specific requirements complicated and prolonged the data application processes, whilst standardized procedures, whenever available, were considered beneficial. A distributed analysis approach in combination with the use of Secure Processing Environments was successfully performed in both use cases. The IT work package of the pilot project developed a technical infrastructure for exchange of metadata and data application information to support the data discovery and application phases of the User’s Journey in the EHDS context.
