# Supplementary material for: The European Health Data Space in communicable diseases surveillance and monitoring of medicines and vaccines: achievements of a pilot project examining the user journey
Source: Eur J Public Health. 2025 Sep 10;35(Suppl 3):iii5–iii10. doi: 10.1093/eurpub/ckaf115 (PMC12420905; doi:10.1093/eurpub/ckaf115)
Supplement: ckaf115_Supplementary_Data [file ckaf115_supplementary_data.docx]

Supplement

Glossary

Health data access body [1]

Defined by article 55 of the EHDS regulation

### Health data holder [1]

Article 2 (t) ‘health data holder’ means any natural or legal person, public authority, agency or other body in the healthcare or the care sectors, including reimbursement services where necessary, as well as any natural or legal person developing products or services intended for the health, healthcare or care sectors, developing or manufacturing wellness applications, performing research in relation to the healthcare or care sectors or acting as a mortality registry, as well as any Union institution, body, office or agency, that has either:

(i) the right or obligation, in accordance with applicable Union or national law and in its capacity as a controller or joint controller, to process personal electronic health data for the provision of healthcare or care or for the purposes of public health, reimbursement, research, innovation, policymaking, official statistics or patient safety or for regulatory purposes; or

(ii) the ability to make available non-personal electronic health data through the control of the technical design of a product and related services, including by registering, providing, restricting access to or exchanging such data;

### Health data user [1]

Article 2 (u) ‘health data user’ means a natural or legal person, including Union institutions, bodies, offices or agencies, which has been granted lawful access to electronic health data for secondary use pursuant to a data permit, a health data request approval or an access approval by an authorised participant in HealthData@EU;

### Secure processing environment [2]

Article 2 (20) ‘secure processing environment’ means the physical or virtual environment and organisational means to ensure compliance with Union law, such as Regulation (EU) 2016/679, in particular with regard to data subjects’ rights, intellectual property rights, and commercial and statistical confidentiality, integrity and accessibility, as well as with applicable national law, and to allow the entity providing the secure processing environment to determine and supervise all data processing actions, including the display, storage, download and export of data and the calculation of derivative data through computational algorithms;

## References

[1] Official Journal of the European Union. Regulation (EU) 2025/327 of the European Parliament and of the Council.

[2] EUR-Lex. Regulation (EU) 2022/868 of the European Parliament and of the Council.
